# Supplementary material for: Prospective Observational Study to Evaluate the Effect of Different Levels of Positive End-Expiratory Pressure on Lung Mechanics in Patients with and without Acute Respiratory Distress Syndrome
Source: J Clin Med. 2020 Jul 31;9(8):2446. doi: 10.3390/jcm9082446 (PMC7463691; doi:10.3390/jcm9082446)
Supplement: Supplementary file 1 [file jcm-09-02446-s001.pdf]

Table 1. Lung mechanics per day and PEEP

**Total of 399 Measurements**				
	PEEP (cmH_2O)	ARDS	Obesity	Controll
Day 1	15	22	18	10
	10	20	18	10
	5	17	16	10
Day 2	15	22	18	9
	10	20	18	9
	5	18	16	9
Day 3	15	16	16	8
	10	16	16	8
	5	15	16	8
